# Measuring the impact of sea surface temperature on the human incidence of *Vibrio sp.* infection in British Columbia, Canada, 1992–2017

**DOI:** 10.1186/s12940-020-00605-x

**Published:** 2020-05-27

**Authors:** Eleni Galanis, Michael Otterstatter, Marsha Taylor

**Affiliations:** 1 British Columbia Centre for Disease Control, 655 W 12th Ave, Vancouver, British Columbia V5Z 4R4 Canada; 2 School of Population and Public Health, University of British Columbia, Vancouver, British Columbia Canada

**Keywords:** Vibrio, *Vibrio parahaemolyticus*, Sea surface temperature, Epidemiology, Climate change

## Abstract

**Background:**

*Vibrio* growth in the environment is related to sea surface temperature (SST). The incidence of human *Vibrio* illness increased sharply in British Columbia (BC) between 2008 and 2015 for unknown reasons, culminating in the largest outbreak of shellfish-associated *Vibrio parahaemolyticus* (Vp) in Canadian history in 2015. Our objective was to assess the relationship between SST and *Vibrio* illness in BC, Canada during 1992–2017 and assess the role of SST and other environmental factors in the 2015 Vp outbreak.

**Methods:**

Cases of *Vibrio* infection reported to the BC Centre for Disease Control during 1992–2017 were used. SST data were obtained from NOAA and NASA. We assessed changes in incidence trend of annual *Vibrio* cases during 1992–2017 using a Poisson regression. We assessed the correlation between annual *Vibrio* cases and the average annual maximum SST using a Spearman rank-order correlation. We modeled the association between weekly Vp case counts, SST and other environmental factors during 2007–2017 using a Poisson regression.

**Results:**

There was a significant increase in *Vibrio* cases between 2008 and 2015 (annual slope = 0.163, *P* < 0.001). Increased *Vibrio* incidence was observed in most El Niño years. There was a significant correlation between annual *Vibrio* cases and maximum SST from 1992 to 2017 (*r* = 0.46, *P* = 0.018). Our model captured observed seasonal variation in shellfish-associated Vp in most years, but underestimated the 2015 Vp outbreak.

**Conclusions:**

*Vibrio* incidence has been increasing concurrently with increasing SST in BC during 2008–2015. The 2015 Vp outbreak was not fully explained by climatic factors and may in part have been associated with other factors. Vp subtyping would be useful in the future to understand the combined effects of SST changes and strain emergence.

## Background

*Vibrio parahaemolyticus* (Vp) is a naturally-occurring bacterium in ocean water worldwide [1]. If ingested, Vp can lead to diarrhea, vomiting, nausea and fever lasting 1–7 days and, rarely, death. The majority of Vp infections are caused by the consumption of raw oysters [1]. Other exposures include the inadvertent swallowing of, or exposure of wounds or external ears to, ocean water.

Sea surface temperature (SST) is thought to be the most important environmental predictor of Vp, with higher temperature leading to higher Vp concentration [2–4]. In British Columbia (BC), Canada, SST was found to be significantly associated with Vp in oysters and with human Vp illness [5]. The vast majority of these Vp infections occurred during the summer months, following the consumption of raw BC oysters [6]. Warm air temperature may also contribute to Vp multiplication in oyster meat post-harvest [7].

The incidence of human Vp illness increased sharply in BC between 2009 and 2015 for unknown reasons [8]. In the summer of 2015, Canada experienced the largest outbreak of Vp cases ever reported and the first outbreak associated with consumption of raw BC oysters since 1997 [9, 10]. Eighty-two people were infected with Vp across the country and 66 (80%) consumed commercially-harvested raw BC oysters. Given that for every case of Vp reported, 92 additional cases are estimated to have occurred in the community, this outbreak may have affected over 7500 Canadians [11]. Sea surface temperature, which was above historical levels at the time, may have played an important role in the outbreak [9]. Following the 2015 outbreak, annual Vp incidence in BC dropped dramatically, returning to levels observed prior to 2009 [8].

The objective of this paper is to analyze the historical relationship between temperature and Vp human incidence in BC and assess its role in the 2015 Vp outbreak.

## Methods

We conducted two analyses: the first was to assess the long-term trends and correlation between annual SST and *Vibrio* illnesses in BC during 1992–2017; the second was to model the association between weekly Vp case counts, temperature and other environmental factors that may have played a role in the 2015 outbreak and subsequent decline in Vp incidence.

### Data

*Vibrio* infection is a reportable disease in BC; a case is defined as a resident of BC with *Vibrio* isolated from stool. BC regional health authority staff interview cases using a standard form to identify the source of illness [12]. The vast majority of *Vibrio* infections in BC are caused by Vp (in 2011–2017, Vp comprised 87.0% of all *Vibrio* infections) [6].

Cases of Vp infection from 2007 to 2017, and cases of all *Vibrio* infection from 1992 to 2017, were extracted from the BC Centre for Disease Control public health information system (Vp specific incidence could not be calculated in early years). Annual incidence rates were calculated using the BC population from BC Stats (www2.gov.bc.ca/gov/content/data/statistics). Weekly numbers of Vp cases where the source of illness was the consumption of shellfish (including all types, raw and cooked, commercial and self-harvested) were used for the regression model.

The study area was the Strait of Georgia, an arm of the Pacific Ocean separating Vancouver Island and the lower mainland of BC, as described elsewhere [5]. To examine long-term trends, historical SST data for 1992 to 2014 were obtained from the National Oceanic and Atmospheric Administration (NOAA) Optimum Interpolation Sea Surface Temperature (OISST) (https://www.ncdc.noaa.gov/oisst). Average annual maximum SST values were calculated from maximum yearly values recorded at three representative points in the study area (Strait of Georgia, 49°52′30.0″N 124°52′30.0″W; west coast of Vancouver Island, 48°22′30.0″N 125°22′30.0″W; east coast of Vancouver Island, 48°52′30.0″N 123°07′30.0″W) and compared against annual counts of all *Vibrio* cases reported in BC during 1992–2017.

For the regression model, SST data for 2007 to 2017 were obtained from the National Aeronautics and Space Administration (NASA) satellite-based Multiscale Ultrahigh Resolution (MUR) product (https://podaac.jpl.nasa.gov/Multi-scale_Ultra-high_Resolution_MUR-SST) [5]. Daily SST values were averaged across all landfile (oyster harvest site) coordinates in the study area and then averaged weekly for April through October 2007–2017. Given that SST values were similar across landfiles (standard deviation of weekly SST = 1.1 °C on average, with a maximum of 2.9 °C), the average weekly value for the study area was used [5]. From these data, annual maximum and minimum SST values were calculated. Daily air temperature data for 2007 to 2017 were obtained (http://climate.weather.gc.ca/). Daily maximum air temperature values from the centrally located Comox A weather station (49°43′00“ N, 124°54’00” W) were summarized as weekly average maximum values.

Annual live oyster production data in dozens were obtained from the Department of Fisheries and Oceans for 2011–2016 and from the BC Ministry of Agriculture for 2007–2010. Monthly live oyster export data in kilograms for 2007–2016 were obtained from the BC Ministry of Agriculture (source: Statistics Canada, CATSNETAnalytics, 2017). These were converted into dozens using dozens = kg*2.2046/4.5 (BC Ministry of Agriculture 2017).

The mean (± standard deviation) time between shellfish harvest and the report of an associated Vp case was 17 days (IQR = 8 days) [5]. Lagged SST and air temperature values for 1, 2 and 3 weeks prior to the week of case report were generated. Previous work in BC has shown that Vp cases are associated with SST values above 14 °C [5]. Therefore, for each year, the first week with mean SST above 14 °C was identified and the cumulative weekly mean SST for each year beginning January 1 and beginning with the first week above 14 °C was calculated.

### Analysis

Significant changes in incidence trend were assessed using a Poisson regression of annual *Vibrio* cases during 1992–2017 fitted using the Joinpoint software [13]. The correlation between annual *Vibrio* cases and average annual maximum SST, and the correlation between weekly Vp cases and average weekly SST, and SST anomaly (current week SST – average SST for that week during previous 3 years), were assessed using a Spearman rank-order correlation.

To model the association between Vp cases, temperature and other factors, Vp case counts for calendar weeks 22–41 (summer period) during 2007–2017 were used. Poisson regressions of weekly shellfish associated Vp cases were developed, including the following predictors: weekly mean SST and maximum air temperature at 1-, 2- and 3-week lags, first week of the year with SST above 14 °C, yearly cumulative SST, annual minimum and maximum SST, annual oyster production volume, and monthly oyster export volume. The fit of models with differing combinations of predictors were compared using the AIC goodness-of-fit statistic. Given the modest sample size for this analysis, we focused on main effects and did not consider two-way or higher order interaction terms. Preliminary modeling showed that oyster availability did not explain variation in Vp counts; hence, annual production and monthly export volumes were excluded from further analysis. We found that weekly SST was a stronger correlate (Spearman correlation: *r* = 0.44, *P* < 0.01) of Vp counts than weekly SST anomaly (Spearman correlation: *r* = 0.10, *P* = 0.15), so only the former was included in our model. We examined other water quality measures (e.g., dissolved oxygen, salinity), but these were not consistently available across our study period.

The best fit model (smallest AIC value) included SST at 1 and 3 week lags from the week of case report, air temperature for lags of 1, 2 and 3 weeks, the cumulative SST for weeks 1–14, and the annual minimum SST.

## Results

**Fig. 1:**
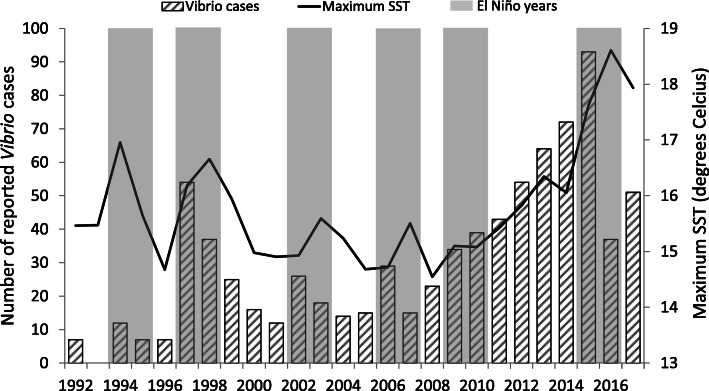
*Vibrio* cases (all types) and yearly maximum sea surface temperature, British Columbia, Canada, 1992–2017. Years with moderate to strong El Niño Southern Oscillation (ENSO) events are shown with vertical grey bands (based on https://origin.cpc.ncep.noaa.gov/products/analysis_monitoring/ensostuff/ONI_v5.php)

No consistent trend in the annual number of reported *Vibrio* cases was detected during 1992–2007 (annual slope = − 0.008, *P* = 0.897) (Fig. 1). After 2007, the number of cases increased every year to a peak of 93 cases in 2015 (2008–2015 annual slope = 0.163 *P* < 0.001). After the outbreak year, numbers dropped to 37 cases in 2016 and 51 in 2017. The change in slope was not significant given the small sample size (2015–2017 annual slope = − 0.347, *P* = 0.235). The annual number of *Vibrio* cases closely followed the average maximum SST (Fig. 1). Every peak in *Vibrio* cases occurred during or near to a period of elevated SST, corresponding to years with moderate to strong El Niño events. The long term increase in SST observed during 2008–2015 coincided with increasing numbers of *Vibrio* cases. Across 1992–2017, the correlation between annual *Vibrio* cases and maximum SST was significant (*r* = 0.46, *P* = 0.018).

**Fig. 2:**
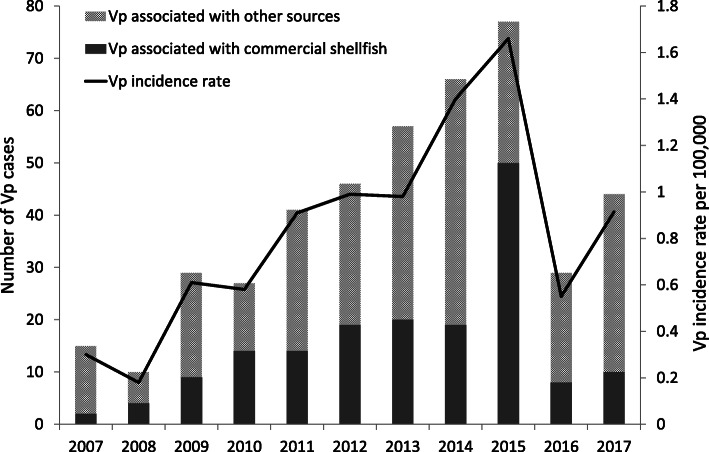
*Vibrio parahaemolyticus* (Vp) cases and incidence rate, British Columbia, Canada, 2007–2017. Other sources include contact with water, travel and unknown source

For *Vibrio parahaemolyticus* (Vp), the highest incidence rate was observed during the outbreak of 2015 at 1.7 cases/100,000 population (Fig. 2). This was mainly driven by an increase in cases associated with the consumption of commercially-harvested BC oysters, from 35% (*N* = 101/291) of Vp cases during 2007–2014 to 65% (*N* = 50/77) in 2015. Vp incidence dropped to 0.9/100,000 by 2017, with an associated 81–84% drop in cases associated with commercial shellfish; indeed, only 8 and 10 reported cases were associated with commercial shellfish in 2016 and 2017, respectively.

**Fig. 3:**
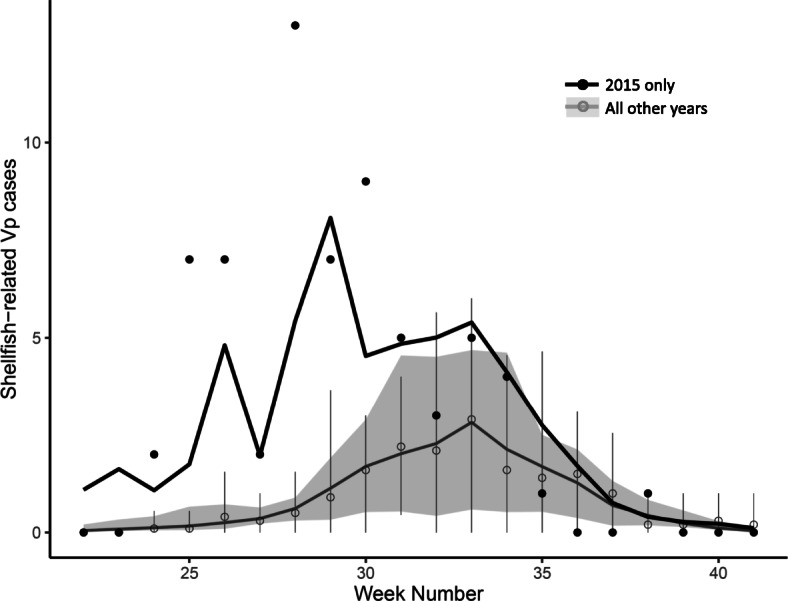
Weekly observed and predicted shellfish-related Vp case counts, BC, 2007–2017. Filled circles represent observed case counts from the outbreak year 2015; open circles and bars represent the mean and 95% CIs of observed case counts across non-outbreak years combined (2007–2014, 2016–2017). The black line shows the model fit for 2015; the grey line and shaded area show the median and minimum to maximum range of model fits for the non-outbreak years

Using sea and air temperature variables, our model captured observed seasonal variation in shellfish-associated Vp in most study years, but notably underestimated the Vp outbreak of 2015 (Fig. 3). Whereas shellfish-associated Vp typically peaked during August (weeks 32–35), with average weekly counts of 2–3 cases, the 2015 outbreak peaked in early July (week 28) with up to 13 cases reported in a single week. Observed and model-predicted case counts differed by less than one case per week on average in non-outbreak years (mean ± SD absolute weekly difference: 0.7 ± 0.8 cases), but differed by almost two cases per week during 2015 (1.6 ± 2.0 cases). At the outbreak peak, the model underestimated the observed data by eight cases.

## Discussion

Our study assessed trends in *Vibrio parahaemolyticus* (Vp) incidence and associated measures of temperature in BC, Canada, before, during and after the 2015 Vp outbreak. The change in annual *Vibrio* cases during 1992–2017 was highly correlated with annual maximum sea surface temperature (SST), including the notable increase during 2008–2015. The peak *Vibrio* sp. incidences observed in 1997, 2002, 2006 and 2015 coincided with periods of elevated SST, reflecting El Niño Southern Oscillations or ENSO occurring in 1997–98, 2002–03, 2006 and 2015–2016 (https://www.esrl.noaa.gov/psd/enso/mei/). Our model based on SST and air temperature also explained the seasonal pattern of Vp incidence during most study years. Air temperature captures the oysters’ exposure to potentially warmer air temperature found in intertidal oyster harvesting, during transport and at retail. However, the 2015 outbreak was not well explained by water and air temperature alone, with many more Vp cases reported than predicted.

Many studies have observed an increase in *Vibrio* sp. in association with warming temperatures globally. The frequency of detection of Vp and other *Vibrio* species has increased in bivalve shellfish on the Pacific and Atlantic coasts of Canada between 2006–9 and 2010–13 [14]. Increasing incidence of *Vibrio* illness has been noted in Europe and the US [15, 16]. In the north Atlantic, the Northern Hemisphere Temperature warming and the Atlantic Multidecadal Oscillation are associated with increasing *Vibrio* present in the water in the last 50 years [17]. In the US, non-cholera *Vibrio* sp. increased from 1999 to 2014; incidence was associated with ENSO conditions and a more rapid increase in incidence was observed at higher latitudes [18].

We suggest that the 2015 outbreak in BC may in part have been associated with climatic factors known to have occurred in BC coastal waters, and in part with other factors such as the introduction of new Vp strains [19]. Climatic factors include the long-term increasing sea temperatures observed between 2008 and 2016, and may also have included the short-term temperature anomalies caused by the 2014–15 northern Pacific blob and the 2015–16 ENSO [9]. The rate of Vp illness can increase over many years in association with slowly increasing SST and large outbreaks can occur over a few months following short-term anomalies in SST [20]. During periods of climate anomalies, the usual relationship between Vp risk and SST may be disrupted; Martinez-Urtaza and colleagues found that during the 1997 El Nino, the risk of Vp infection in Peru was no longer associated with SST, but rather with sea height anomaly and heat content above 20C, parameters that were not available in BC [21].

Beyond climatic factors, the introduction of new Vp strains into BC coastal waters may play a key role in Vp outbreaks. New strains may be imported through the discharge of ballast water from large ships during periods of warm weather, or through the introduction of warm water and invasive zooplankton transported from other regions [21, 22]. Various Vp strains circulate in the Pacific Northwest, including (ST)36, one of the most virulent strains [23, 24]. Unfortunately, Vp isolates from 2015 were not available to assess strain variation.

The rapid drop in Vp incidence after the 2015 outbreak was mainly due to a decrease in cases associated with commercially-harvested shellfish. Following the outbreak, governmental, scientific and industry stakeholders in BC collaborated to identify gaps and implement actions to control Vp in commercial shellfish. These control measures may have contributed to the decrease in Vp incidence, as was also observed following the 1997 Pacific Northwest outbreak and the 2004 Alaska outbreak [6, 22].

Washington (WA) State also reported increasing *Vibrio* sp. incidence between 2008 and 2014 (high of 1.3/100,000) [25]. In 2015, the WA Department of Health implemented a *Vibrio* Control Plan to determine the risk of shellfish growing areas and appropriate harvest requirements [26]. Although WA coastal waters were exposed to the same environmental conditions and presumably the same Vp strains as BC, they did not experience an outbreak in 2015 (incidence of 1.0/100,000). This suggests that, although temperature is an important determinant of Vp growth, Vp may be controlled through mitigation measures such as those implemented for commercial shellfish in WA in 2015 and in BC in 2016.

This is an ecological study where changes in environmental factors were associated with population-level changes in Vp incidence. The observed changes in incidence cannot be directly attributed to a specific cause. We attempted to account for factors that could affect Vp incidence, namely SST, air temperature and product availability; however, we were limited by a lack of data for certain factors. Oyster consumption, for example, is not directly measured in BC. Instead, we used oyster production and oyster exports together as a proxy for oyster consumption. These data were available only at broader time scales (annual production, monthly exports) and it is likely that production varied during the year, particularly with increases during the summer. This may explain part of the seasonal increase in Vp incidence during 2015 and why our model underestimated the number of cases during the outbreak peak. We did not have reliable data on Vp in ocean water or in oysters. However, Konrad et al found that SST was an excellent predictor of the risk of Vp in oysters and of Vp illness in BC [5]. We considered other environmental parameters (e.g., ocean salinity and oxygen), but these were not consistently available in our study area for all study years. We did not have Vp strain data, given that typing was not routinely conducted during the study period.

## Conclusions

*Vibrio* incidence was strongly related to sea surface temperature and, in our model, both sea and air temperatures were important predictors of Vp incidence in non-outbreak years. However, temperature did not fully explain the 2015 outbreak in BC and other factors may have contributed. Following the outbreak, control measures were implemented along the shellfish food chain which may have contributed to a decrease in Vp incidence while SST remained high. Vp subtyping would be useful in the future to understand the role of different strains in human illness, and the combined effects of changes in SST and strain emergence. Now that Vp incidence has decreased in BC, it is essential to maintain control measures and ongoing awareness to avoid the increase in *Vibrio* illness that can occur with global warming.

## Data Availability

The environmental datasets analysed during the current study are available here: • National Oceanic and Atmospheric Administration Optimum Interpolation Sea Surface Temperature (https://www.ncdc.noaa.gov/oisst) • National Aeronautics and Space Administration satellite-based Multiscale Ultrahigh Resolution product (https://podaac.jpl.nasa.gov/Multi-scale_Ultra-high_Resolution_MUR-SST) • Environment and Climate Change Canada (http://climate.weather.gc.ca/) The human illness datasets analysed during the current study are not publicly available due patient confidentiality concerns but are available for reasonable requests from the BC Centre for Disease Control through http://www.bccdc.ca/about/accountability/data-access-requests.

## References

[1] Control of Communicable Diseases Manual. 20th ed. Heymann DL, editor. Washington DC: APHA; 2015.

[2] Cook DW, Bowers JC, DePaola A (2002). Density of Total and pathogenic (tdh+) Vibrio parahaemolyticus in Atlantic and Gulf Coast Molluscan shellfish at harvest. J Food Prot.

[3] Haley BJ, Kokashvili T, Tskshvediani A, Janelidze N, Mitaishvili N, Grim CJ (2014). Molecular diversity and predictability of *Vibrio parahaemolyticus* along the Georgian coastal zone of the Black Sea. Front Microbiol.

[4] Parveen S, Hettiarachchi KA, Bowers JC, Jones JL, Tamplin ML, McKay R (2008). Seasonal distribution of total and pathogenic *Vibrio parahaemolyticus* in Chesapeake Bay oysters and waters. Int J Food Microbiol.

[5] Konrad S, Padurao P, Romero-Barrios P, Henderson S, Galanis E (2017). Remote sensing measurements of sea surface temperature as an indicator of Vibrio parahaemolyticus in oyster meat and human illnesses. Environ Health.

[6] Khaira G, Galanis E (2007). Descriptive epidemiology of Vibrio parahaemolyticus and other Vibrio species infections in British Columbia: 2001-2006. Can Comm Dis Rep..

[7] Gooch JA, DePaola A, Bowers J, Marshall DL (2002). Growth and survival of Vibrio parahaemolyticus in postharvest American oysters. J Food Prot.

[8] BCCDC. Reportable Disease Dashboard. 2018 [cited 2018 Mar 16]. Available from: http://www.bccdc.ca/health-info/disease-system-statistics/reportable-disease-dashboard.

[9] Taylor Marsha, Cheng Joyce, Sharma Davendra, Bitzikos Olga, Gustafson Reka, Fyfe Murray, Greve Richard, Murti Michelle, Stone Jason, Honish Lance, Mah Victor, Punja Nisha, Hexemer April, McIntyre Lorraine, Henry Bonnie, Kendall Perry, Atkinson Robin, Buenaventura Enrico, Martinez-Perez Amalia, Galanis Eleni, Team the Outbreak Invesitigation (2018). Outbreak of Vibrio parahaemolyticus Associated with Consumption of Raw Oysters in Canada, 2015. Foodborne Pathogens and Disease.

[10] Fyfe M, Yeung ST, Daly P, Schallie K, Kelly MT, Buchanan S (1997). Outbreak of Vibrio Parahaemolyticus related to raw oysters in British Columbia. Can Comm Dis Rep.

[11] Thomas MK, Murray R, Flockhart L, Pintar K, Pollari F, Fazil A (2013). Estimates of the burden of foodborne illness in Canada for 30 specified pathogens and unspecified agents, circa 2006. Food Pathog Dis.

[12] BCCDC. Vibrio Infection Case Report Form. 2017 [cited 2018 March 16]. Available from: http://www.bccdc.ca/health-professionals/professional-resources/surveillance-forms.

[13] Joinpoint Regression Program. Version 4.1.1 ed. Statistical Research and Applications Branch: National Cancer Institute; August 2014.

[14] Banerjee SK, Rutley R, Bussey J. Diversity and dynamics of the canadian coastal vibrio community: an emerging trend detected in the temperate regions. J Bacteriol. 2018;200(15):200e00787-17. 10.1128/JB.00787-17 PMC6040189 29735763

[15] Baker-Austin Craig, Trinanes Joaquin A., Taylor Nick G. H., Hartnell Rachel, Siitonen Anja, Martinez-Urtaza Jaime (2012). Emerging Vibrio risk at high latitudes in response to ocean warming. Nature Climate Change.

[16] Newton A, Kendall M, Vugia DJ, Henao OL, Mahon BE (2012). Increasing rates of vibriosis in the United States, 1996-2010: review of surveillance data from 2 systems. Clin Infect Dis.

[17] Vezzulli L, Grande C, Reid PC, Helaouet P, Edwards M, Hofle MG (2016). Climate influence on Vibrio and associated human diseases during the past half-century in the coastal North Atlantic. Proc Natl Acad Sci U S A.

[18] Logar-Henderson C, Ling R, Tuite AR, Fisman DN (2019). Effects of large-scale oceanic phenomena on non-cholera vibriosis incidence in the United States: implications for climate change. Epidemiol Infect.

[19] Government B. Change in Sea Surface Temperature in B.C. (1935-2014) 2017 [cited 2020 Mar 19]. Available from: http://www.env.gov.bc.ca/soe/indicators/climate-change/sea-surface-temperature.html.

[20] Martinez-Urtaza J, Bowers JC, Trinanes J, DePaola A (2010). Climate anomalies and the increasing risk of Vibrio parahaemolyticus and Vibrio vulnificus illnesses. Food Res Int.

[21] Martinez-Urtaza J, Huapaya B, Gavilan RG, Blanco-Abad V, Ansede-Bermejo J, Cadarso-Suarez C (2008). Emergence of Asiatic Vibrio diseases in South America in phase with El Nino. Epidemiology..

[22] McLaughlin JB, DePaola A, Bopp CA, Martinek KA, Napolilli NP, Allison CG (2005). Outbreak of Vibrio parahaemolyticus gastroenteritis associated with Alaskan oysters. N Engl J Med.

[23] Banerjee SK, Kearney AK, Nadon CA, Peterson CL, Tyler K, Bakouche L (2014). Phenotypic and genotypic characterization of Canadian clinical isolates of Vibrio parahaemolyticus collected from 2000 to 2009. J Clin Microbiol.

[24] Martinez-Urtaza J, van Aerle R, Abanto M, Haendiges J, Myers RA, Trinanes J (2017). Genomic variation and evolution of *Vibrio parahaemolyticus* ST36 over the course of a transcontinental epidemic expansion. mBio.

[25] WADOH. Washington State Communicable Disease Report 2016. 2017 [cited 2018 March 16]. Available from: https://www.doh.wa.gov/Portals/1/Documents/5100/420-004-CDAnnualReport2016.pdf.

[26] WADOH. Vibrio Control Plan Rule Requirements. 2015 [cited 2018 March 16]. Available from: https://www.doh.wa.gov/CommunityandEnvironment/Shellfish/CommercialShellfish/VibrioControlPlan.

